# Chemical characterization and assessment of antioxidant potentiality of *Streptocaulon sylvestre* Wight, an endangered plant of sub-Himalayan plains of West Bengal and Sikkim

**DOI:** 10.1186/s12906-015-0629-0

**Published:** 2015-04-08

**Authors:** Priyankar Dey, Sandipan Ray, Mousumi Poddar Sarkar, Tapas Kumar Chaudhuri

**Affiliations:** Cellular Immunology Laboratory, Department of Zoology, University of North Bengal, Siliguri, 734013 West Bengal India; Chemical Signal and Lipidomics Laboratory, Department of Botany, University of Calcutta, Kolkata, 700019 India

**Keywords:** Antioxidant, Flavonoid, Free radical, GC-MS, Haemolytic, HPLC, Iron chelation, Lipid peroxidation, Phenolic, Streptocaulon

## Abstract

**Background:**

*S. sylvestre* Wright is an extremely rare plant, found only in the sub-Himalayan Terai region of West Bengal and neighboring Sikkim foot-hills. The plant has never been evaluated for any pharmaceutical properties. The phytochemical status of the plant is still unknown. Therefore, the aim of the study was to explore the antioxidant and free radical scavenging activities and analysis of bioactive compounds present in *S. sylvestre.*

**Methods:**

*S. sylvestre* methanolic extract (SSME) was evaluated for different free radical scavenging activities such as hydroxyl radical, nitric oxide, singlet oxygen, hypochlorous acid, peroxynitrite, superoxide radical and hydrogen peroxide scavenging etc. Iron chelating capacity and inhibition of lipid peroxidation were studied in addition to the assessment of haemolytic activity and erythrocyte membrane stabilizing activity (EMSA). Chemical characterization of SSME were performed by high performance liquid chromatography (HPLC) and gas chromatography–mass spectrometry (GC-MS).

**Results:**

The results indicate that SSME possess potent antioxidant activity with IC_50_ value of 113.06 ± 5.67 μg/ml, 63.93 ± 4.16 μg/ml and 142.14 ± 6.13 μg/ml for hydroxyl radical, superoxide radical and hypochlorous acid, respectively. HPLC analysis revealed presence of different phenolic secondary metabolites such as gallic acid, ferulic acid, p-coumaric acid, syringic acid, myricetin, quercetin etc. GC-MS analysis displayed the predominance of γ-sitosterol, vitamin E and squalene in SSME.

**Conclusion:**

The present study provides a convincing evidence that *S. sylvestre* not only possess potent antioxidant activity but also can be used as a source of natural bioactive phytochemicals in the future.

**Electronic supplementary material:**

The online version of this article (doi:10.1186/s12906-015-0629-0) contains supplementary material, which is available to authorized users.

## Background

**Figure 1:**
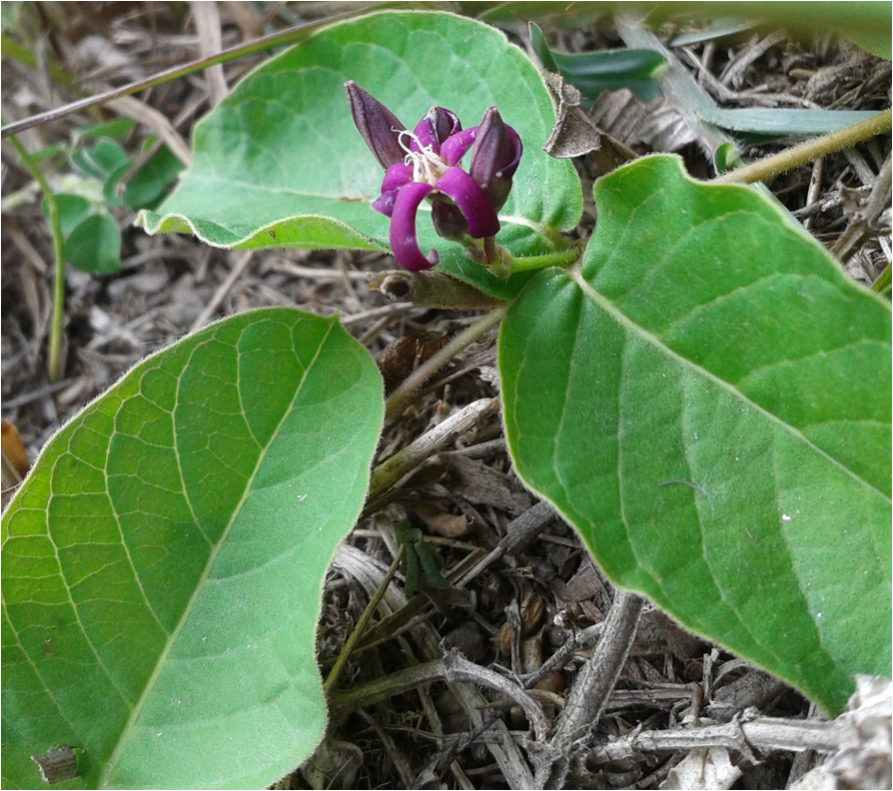
**Picture of** ***Streptocaulon sylvestre*** **Wright.** Picture was taken at the garden of Department of Zoology, University of North Bengal.

The genus *Streptocaulon* belonging to the family Apocynaceae (previously Asclepiadaceae) having extensive distribution in tropical Asia. Venter and Verhoeven described nine distinct species under the genus *Streptocaulon* [1]. However, according to ‘The Plant List’, 23 plants are enlisted under *Streptocaulon* with 6 accepted, 10 synonyms and 7 unresolved names [2] including S. *sylvestre* Wight (Figure 1) which is presently under review by the ‘World Checklist of Selected Plant Families’ (WCSP). *S. sylvestre* is an extremely rare plant (Additional file 1). According to present literature survey, distribution of the plant is restricted only to very selective parts of the earth i.e. in the sub-Himalayan plains of West Bengal and foot-hills of Sikkim Himalaya in India [3,4]. The plant was initially collected by Hamilton (Ham. Herb. No. 763) in 1809 form Sannyasikata at Rajgunj block in Jalpaiguri district (West Bengal, India) and subsequently named by Wright in 1834 [3]. Presently the plant is only reported to grow in several patches in different open fields within the campus of University of North Bengal (NBU) [3], including the garden of Department of Zoology. The University administration has very recently take a radical initiative for *in situ* conservation of the plant growing in several patches on a land near the Mathematics department of NBU and given a local name ‘Uttara’ to the plant. In addition, our lab has maintained and taking care of different patches of *S. sylvestre* growing in the garden our Zoology department. At present, best to our knowledge, NBU is the only place in entire world where the plant can be found growing in its natural habitat.

Very limited scientific information is available on this rare plant because of it’s non-availability. PubMed search revealed no available information in the global database however, Google search resulted in around 2,640 hits, but most of them were actually hits on different plants of the same genus. Several studies have been conducted on the medicinal properties of other plants of the genus *Streptocaulon. In vitro* antioxidant activity of *S. juventas* and *S. baummi* were studied previously [5,6]. Various extracts and bioactive cardiac glycoside isolated from *S. juventas* displayed anti-proliferative activity against different cancer cell-lines [7-12]. Similarly, bioactive glycosides were isolated from *S. griffithii* which revealed potent anti-cancer properties [13-15]. Wessjohann and his group reported anti-cancer property of cardenolides isolated from *S. tomentosum* [16]. Unfortunately, no report is available either regarding the bioactivity of *S. sylvestre* or regarding its medicinal value in the ethnopharmacological literatures.

Pharmacological activities of *S. sylvestre* were evaluated by studying antioxidant and free radical scavenging activities in addition to assessment of iron chelation, haemolytic activity, inhibition of lipid peroxidation and erythrocyte membrane stabilizing activity (EMSA) in this first hand pilot study. Besides, chemical characterization were performed by spectrophotometric quantification of total phenolic and flavonoid contents and by chromatographic methods.

## Methods

### Chemicals and solvents

All the reagents were procured from HiMedia Laboratories Pvt. Ltd. (Mumbai, India), unless otherwise indicated. 6-hydroxy-2,5,7,8-tetramethychroman-2-carboxylic acid (Trolox) was obtained from Fluka, Switzerland. Hydrogen peroxide was obtained from SD fine-chem Ltd (Mumbai, India). HPLC standards were procured form Sigama Aldrich (USA) and ChromaDex (USA). HPLC grade solvents were obtained from Sisco Research Laboratories Pvt Ltd (India). Milli-Q ultrapure water from the departmental facility was used in all the experiments.

### Plant material

*S. sylvestre* plant grows in different small patches in the ground. The plant was previously identified and authenticated by plant taxonomist Prof. A. P. Das of Dept. of Botany. Voucher specimen was also previously stored in the herbarium of NBU. Around 200 g whole plant including root, stem and leaves were collected from 25 ± 2 different patches from the garden of Dept. of Zoology, NBU (26°42'35.3"N, 88°21'08.4"). The sampling was performed in random in case of patch selection and also plant part (root, stem, root) selection. All the plant samples were collected in a single day (September 6th, 2014) and in a single sampling event.

### Sample preparation

The fresh and disease free plant material was washed twice with double distilled water, shade dried at room temperature for 21 days and then grinded to powder. The powder (100 g) was mixed with 100% methanol (1000 ml) and kept in a shaking incubator (160 rpm) for 18 h at 37°C. Then the mixture was centrifuged at 5000 rpm for 15 minutes. The pellet was resuspended in 100% methanol (1000 ml) and kept overnight at the shaking incubator as previous. The supernatant liquid was collected from both the phases and filtered. The resultant filtrate was concentrated in a rotary evaporator under reduced pressure. The concentrated *S. sylvestre* methanolic extract (SSME) was lyophilized and stored at −20°C until further use. The final yield was 6% of the initial powdered sample.

### Collection of brain samples and blood

Blood from Swiss albino mouse was collected in a EDTA containing tube by puncturing the heart under proper anesthesia. The blood samples were used for EMSA and haemolytic activity assay. Lipid peroxidation assay was performed using the brain of the same mouse. All the experiments were approved by the ethical committee of the Department of Zoology, NBU and performed in accordance with the legislation for the protection of animals used for scientific purposes.

### Trolox equivalent antioxidant capacity (TEAC)

TEAC of SSME was assayed on the basis of the ability of SSME to scavenge ABTS^•-^ under a duration of 6 min, which was compared to standard trolox [17]. The percentage inhibition of absorbance was calculated and plotted as a function of concentration of trolox and SSME to determine the TEAC. TEAC was calculated by dividing the gradient of plot for SSME by the gradient of plot for trolox.

### DPPH radical scavenging assay

DPPH nitrogen radical scavenging assay was performed on the basis of reduction of 2,2-diphenyl-1-picrylhydrazyl (DPPH) recorded at 517 nm according to a standard method [18]. Ascorbic acid was used as standard.

### Hydroxyl radical scavenging assay

Hydroxyl radical (OH^•^) was generated through Fenton reaction of Fe^3+^-ascorbate-EDTA-H_2_O_2_ system and subsequently condensation of the degradation product of 2-deoxyribose with thiobarbituric acid (TBA) was estimated at 532 nm by the standard method of Elizabeth and Rao [19]. The classical OH^•^ scavenger mannitol was used as standard.

### Superoxide radical scavenging assay

Superoxide radical (O_2_^•−^), generated by the non-enzymatic combination of phenazine methosulfate (PMS) and reduced nicotinamide adenine dinucleotide (NADH), was measured by the reduction of nitro blue tetrazolium (NBT) to purple-colored formazan using a previously standardized method [18]. Quercetin was used as standard.

### Nitric oxide (NO) radical scavenging assay

At physiological pH, NO was generated through the reaction between aqueous sodium nitroprusside (SNP) and oxygen, which was quantified by the Griess Ilosvoy reaction [20]. The pink azo-dye generated due to diazotization of nitrite ions with sulphanilamide and subsequent coupling with N-(1-naphthyl) ethylenediamine dihydrochloride (NED) was measured at 540 nm using Curcumin as standard.

### Hydrogen peroxide scavenging assay

Hydrogen peroxide scavenging capacity of SSME was studied by FOX reagent method in which oxidation product of Fe^2+^ binds to xylenol orange. The absorbance was measured at 560 nm [21]. Sodium pyruvate was used as standard.

### Peroxynitrite scavenging activity

Previously described method of Beckman *et al.* [22] was followed to generate peroxynitrite (ONOO^−^). Further, Evans blue bleaching assay, as described by Bailly *et al.* [23], was followed to assay the ONOO^−^ scavenging activity of SSME at 611 nm. Gallic acid was used as standard.

### Singlet oxygen scavenging assay

Singlet oxygen (^1^O_2_) was generated by the reaction of sodium hypochloride (NaOCl) and H_2_O_2_, and the ^1^O_2_ scavenging activity of SSME was measured by monitoring the bleaching of N,N-dimethyl-4-nitrosoaniline (RNO) at 440 nm following a previously standardized method [18]. Lipoic acid was used as standard.

### Hypochlorous acid scavenging assay

Hypochlorous acid (HOCl) was freshly generated by a reaction between NaOCl and H_2_SO_4_ at pH 6.2. The HOCl scavenging activity of SSME was measured at 404 nm by monitoring the decrease in absorbance of catalase following the method of Aruoma and Halliwell [24]. Ascorbic acid was used as standard.

### Measurement of reducing power

The reducing capacity of SSME was measured at 700 nm following a standard method [25] where higher absorbance represents higher reducing power. Ascorbic acid was used as standard.

### Lipid peroxidation inhibition assay

Inhibition of lipid peroxidation capacity of SSME was assayed by studying the inhibition of OH^−^ catalyzed malondialdehyde (MDA) production from the polyunsaturated fatty acid (PUFA) in the mice brain samples. The decrease in absorbance was measured at 532 nm and trolox was used as standard [26].

### Fe^2+^ chelation

Iron chelation activity of SSME was evaluated by measuring the decrease of intensity of violet complex, generated by coupling of Fe^2+^ and ferrozine at 562 nm following the method described by Haro-Vicente *et al*. [27]. EDTA was used as standard.

### Total antioxidant activity (TAA)

TAA was studied at 695 nm following the method of by Prieto *et al*. [28] observing the reduction of Mo^6+^ to Mo^5+^ by SSME. Ascorbic acid was used as standard.

### Quantification of total phenolic and flavonoid content

Total phenolic and flavonoid content of SSME was estimated at 725 nm and 510 nm, respectively by the previously standardized methods [18]. The phenolic and flavonoid contents were calculated from gallic acid (GA; R^2^ = 0.9708) and quercetin (QC; R^2^ = 0.9891) standard curve, respectively.

### Erythrocyte membrane stabilizing activity (EMSA)

EMSA of SSME was assayed using a standard method of Navarro *et al.* [29]. Quercetin was taken as standard. The erythrocyte membrane stabilizing activity was measured by the following equation:

$$ \%\ \mathrm{of}\ \mathrm{protection} = \left(\mathrm{A}\mathrm{s}\ /\ \mathrm{Ab}\right) \times 100 $$

Where, As and Ab are the absorbance value of SSME/standard and blank, respectively.

### *In vitro* haemolytic activity

Haemolytic activity of SSME was measured using mice blood by the measurement of released haemoglobin at 540 nm following the method of Kalaivani *et al.* [30]. Triton-X100 (0.1%) was used as standard.

### HPLC analysis

SSME was subjected to Bligh and Dyer’s method [31] to remove the lipid contents. The upper methanolic fraction was used in next steps for elimination of protein and for extraction of secondary metabolites. Chilled acetone was added to the methanolic fraction in the ratio of 4:1 (v/v) and kept for 1 h at −20°C. The solution was then centrifuged twice for 15 min. at 15,000 × *g* (4°C). The pellet containing protein was discarded and the supernatant was subjected to thin layer chromatography (TLC) on a silica gel plate using 10% acetic acid in chloroform as solvent. The corresponding bands of phenolic, flavonoids and methylphenol were eluted by acetonitrile after detection with 20% w/v Na_2_CO_3_ and diluted Folin-Ciocaltaeu reagent (1:3). The solution was then analyzed using HPLC (Agilent, USA) having Zorbax SB-C18 column (4.6 × 150 mm, 3.5 micron) and equipped with Diod Array Detector. Gradient concentration of mobile phase A - methanol (M) and B - water (W) with 0.02% H3PO4 were 25% A + 75% B for 5 min, 30% A + 70% for 10 min, 45% A + 55% for 30 min and 80% A + 20% B for 45 min. The injection volume was 20 μl and the flow rate was kept at 0.4 ml/min in every cases. Analytes were scanned in 275 nm. The peaks were identified by comparing the relative retention time (RRT), co-chromatography and spectral patterns obtained from the standards of phenolic acids, flavonoids and methylphenol with a concentration of 0.05 mg/ml and run under the same condition. The analytes were estimated following external method by calibrating against response factor obtained from the known amount of authentic compounds with proper validation criteria. The concentration of the analytes were expressed in μg/g as calculated from the dry weight (dw) of SSME initially measured for the extraction process.

### GC-MS analysis

SSME was separately dissolved in n-hexane and dichloromethane (DCM) and the mixtures were centrifuged thrice at 12000 rpm for 15 min. The two clear supernatant was used for GC-MS analysis for identification of different classes of phytochemicals. Agilent 5975C GC-MS system (Agilent Technologies, USA) attached with HP-5 ms Capillary Column (30 m × 0.25 mm i.d. × 0.25 μm film thickness) and equipped with inert MSD triple axis mass detector conditioned at ion trap 200°C, transfer line 280°C, electron energy 70 eV (vacuum pressure- 2.21e-0.5 torr) was used for analysis. The carrier gas was helium at a flow rate of 1 ml/min. 2 μl sample was injected in a splitless mode. The column temperature was set at 60°C for 1 min. followed by 5°C/ min upto 250°C. The major and essential compounds in SSME were identified by their retention times and mass fragmentation patterns using Agilent Chem Station integrator and the database of National Institute Standard and Technology (NIST) with a MS library version 2010 and by analyzing MS fragmentation patterns.

### Statistical analysis

All qualitative data are reported as the mean ± SD of six measurements. Statistical analysis was performed by paired t-tests using KyPlot version 2.0 beta 15 (32 bit). P < 0.05 was considered significant. Percentage of inhibition/scavenging was calculated by the formula $=\frac{X0-X1}{X0} \times 100$, where X0 was the absorbance of the control and X1 was the absorbance in the presence of the samples and standard. The half maximal inhibitory concentration (IC_50_) values were calculated by the formula $Y=\frac{A1}{X+A1} \times 100$, where A1 = IC_50_, Y = response (Y = 100% when X = 0), X = inhibitory concentration. GC-MS analysis for identification of phytocompounds was done from three biological replicates for confirmation.

## Results

### Antioxidant and free radical scavenging activity

**Figure 2:**
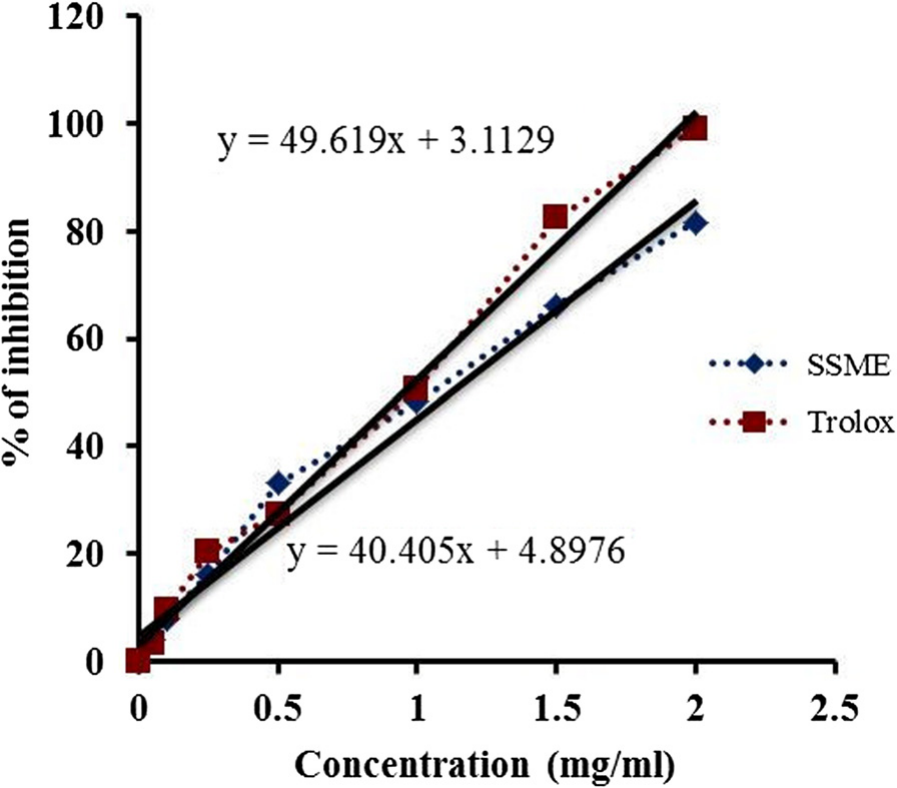
**Trolox equivalent antioxidant capacity of SSME.** The percentage inhibition was plotted against the concentration of sample. All data are expressed as mean ± S.D. (n = 6).

**Figure 3:**
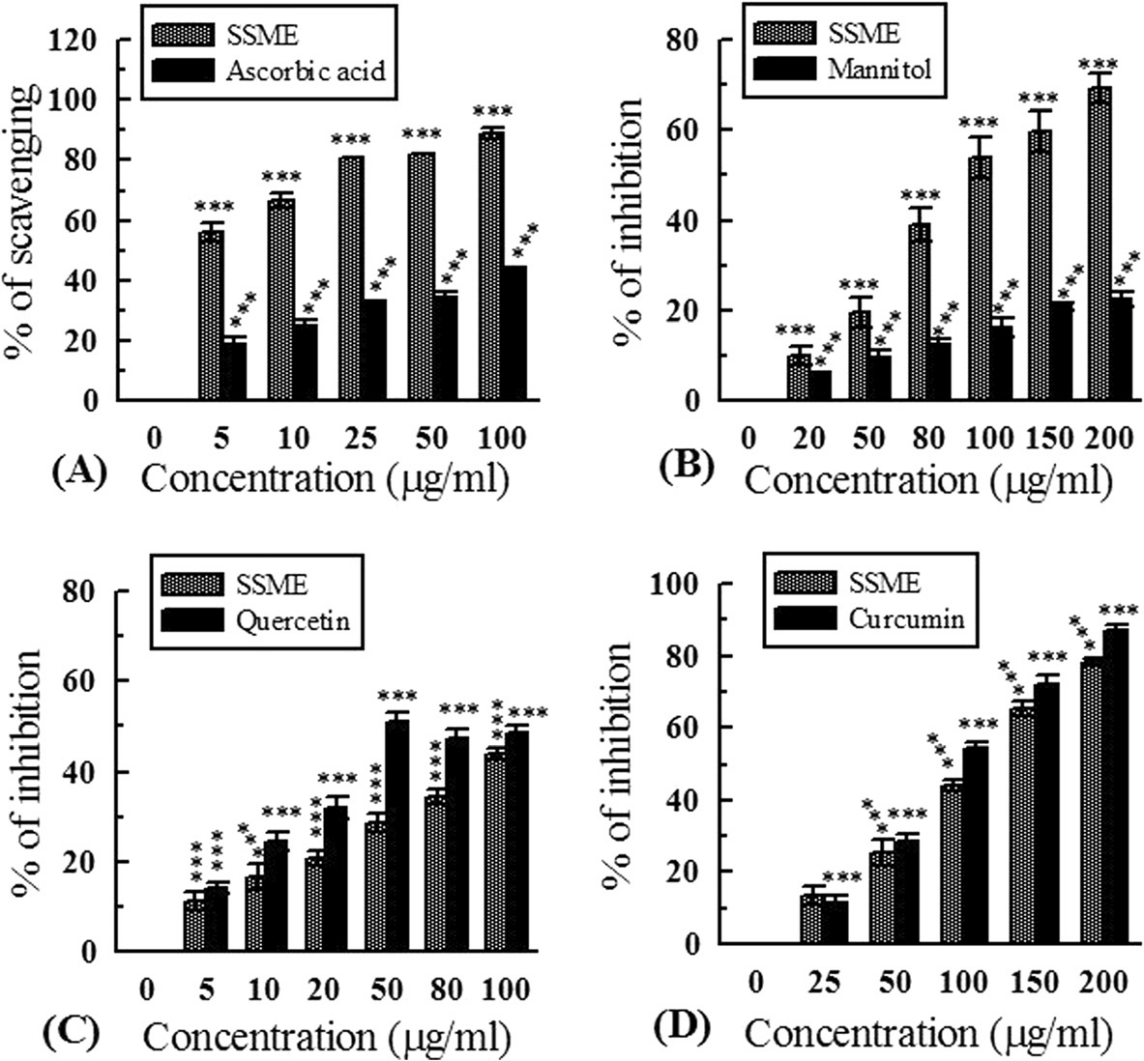
**Antioxidant activity of** ***S. sylvestre*** **. (A)** DPPH radical scavenging activity; **(B)** Hydroxyl radical scavenging activity; **(C)** Superoxide radical scavenging activity; **(D)** Nitric oxide scavenging activity. ***p < 0.001 vs 0 μg/ml.

**Figure 4:**
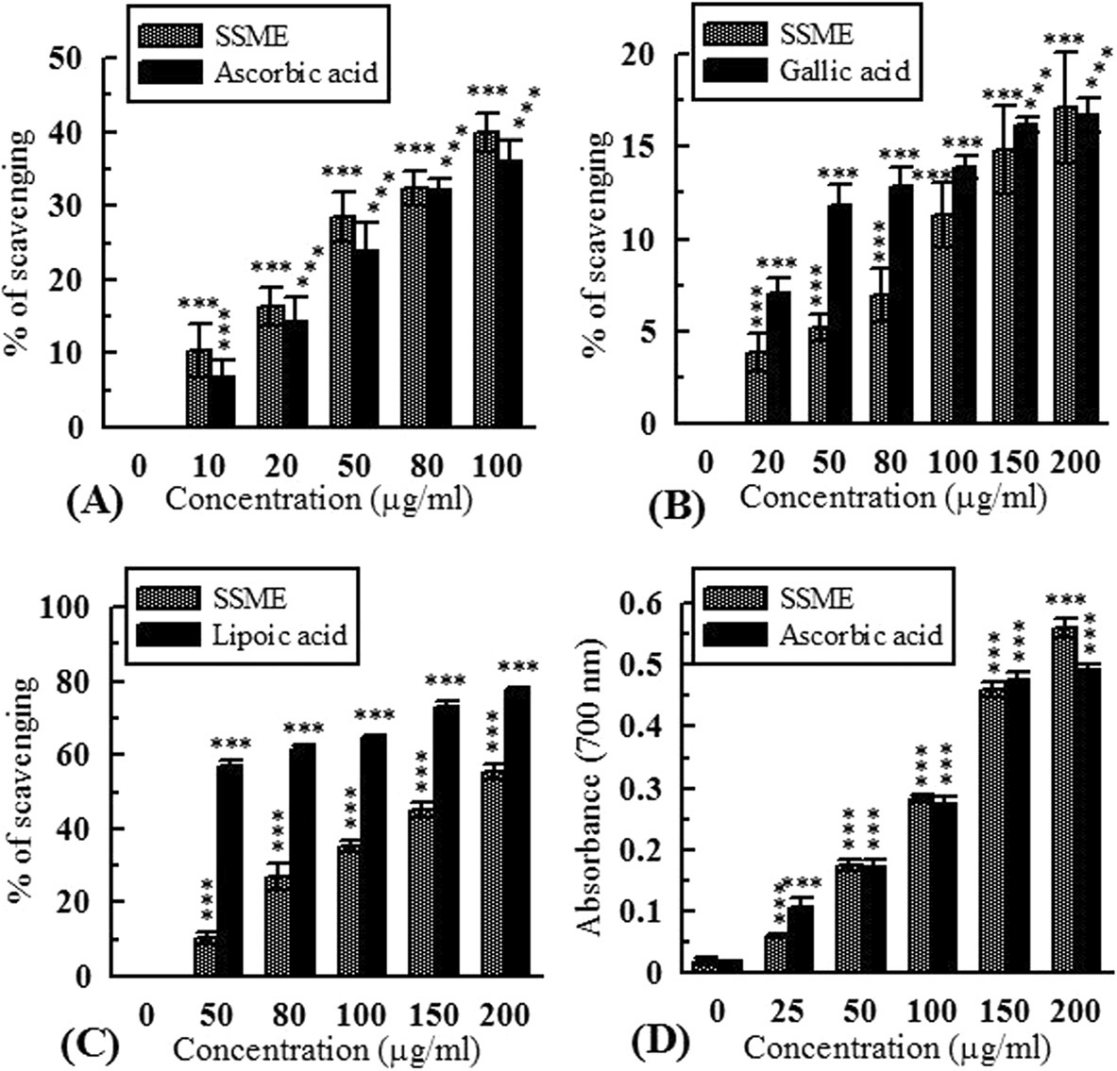
**Antioxidant activity of** ***S. sylvestre.*** **(A)** Hypochlorous acid scavenging activity; **(B)** Peroxynitrite scavenging activity; **(C)** Singlet oxygen scavenging activity; **(D)** Reducing power. ***p < 0.001 vs 0 μg/ml.

**Figure 5:**
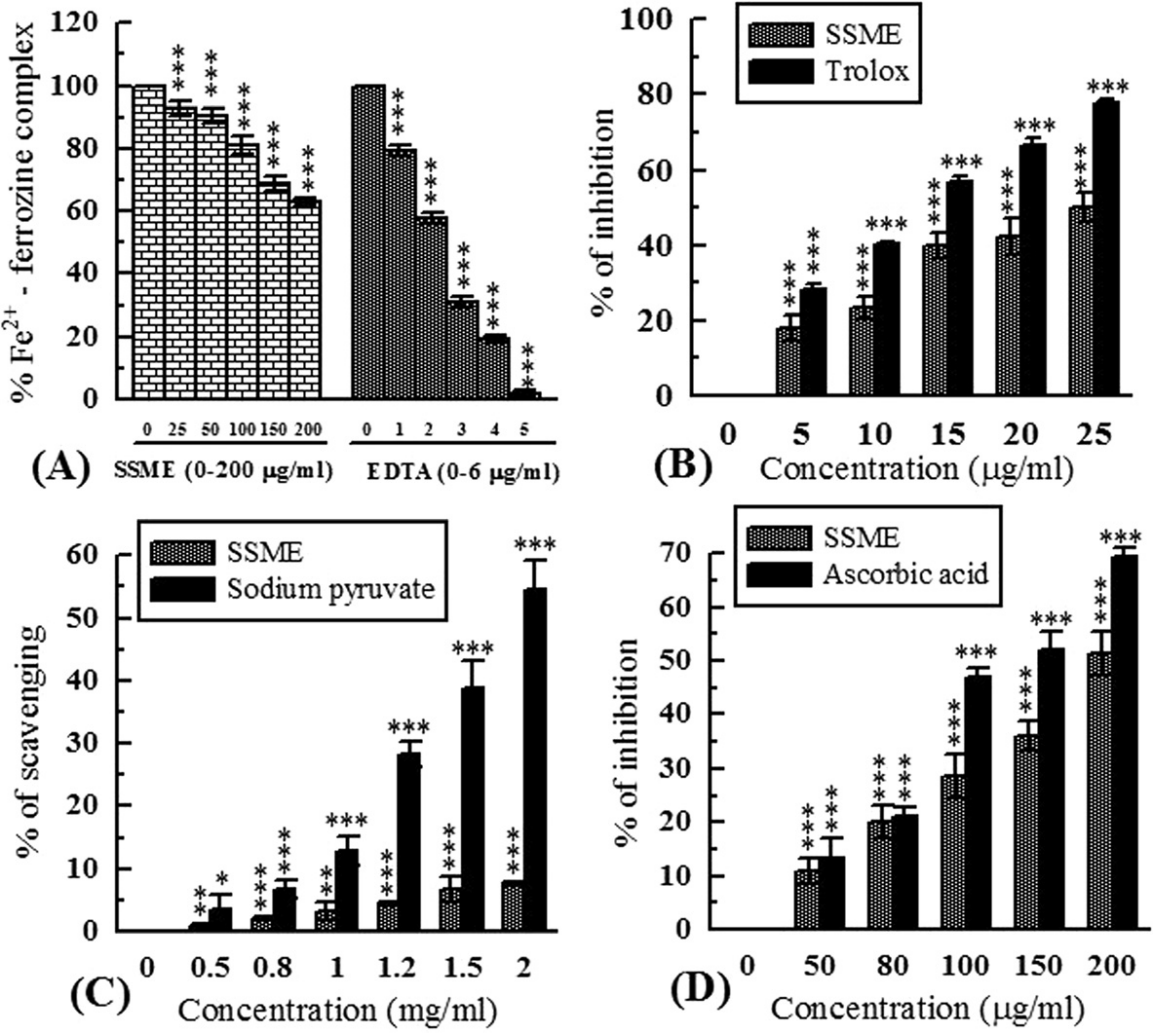
**Antioxidant activity of** ***S. sylvestre.*** **(A)** Iron chelation activity; **(B)** Inhibition of lipid peroxidation; **(C)** Hydrogen peroxide scavenging activity; **(D)** Total antioxidant activity. **P < 0.01 and ***P < 0.001 vs 0 μg/ml.

Antioxidant and free radical scavenging capacities of SSME and corresponding standards are demonstrated in Figures 2, 3, 4, and 5. At 2 mg/ml concentration, the amount of inhibition of ABTS^•-^ by trolox and SSME were 99.18 ± 0.19% and 81.69 ± 1.87%, respectively (Figure 2). The TEAC value of SSME was calculated to be 0.81 ± 0.006. The half maximal inhibitory concentration of SSME is significantly lower than the corresponding standards in DPPH, OH^•^, O_2_^•−^ and HOCl scavenging assays as displayed in Table 1.

**Table 1:** **Half maximal inhibitory concentration (IC**_**50**_ **) of SSME and standards for different antioxidant and free radical scavenging assays**

**Parameters**	**SSME**	**Standard**
DPPH	4.91 ± 0.57**	83.80 ± 7.27 (ascorbic acid)
Hydroxyl radical	113.06 ± 5.67***	589.06 ± 46.57 (mannitol)
Superoxide anion	63.93 ± 4.16***	121.41 ± 5.72 (quercetin)
Nitric oxide	101.62 ± 4.70***	80.46 ± 3.10 (curcumin)
Hydrogen peroxide	25.48 ± 3.13***	3.17 ± 0.14 (sodium pyruvate)
Peroxynitrite	0.90 ± 0.07**	0.73 ± 0.02 (gallic acid)
Singlet oxygen	196.36 ± 12.58***	48.40 ± 1.81 (lipoic acid)
Hypochlorous acid	142.14 ± 6.13*	165.91 ± 16.31 (ascorbic acid)
Total antioxidant activity	252.74 ± 17.54***	150.01 ± 7.31 (ascorbic acid)
Iron chelation	361.80 ± 12.05***	1.45 ± 0.04 (EDTA)
Lipid peroxidation	26.07 ± 3.15***	11.11 ± 0.22 (trolox)

Units in μg/ml, except H_2_O_2_, peroxynitrite scavenging and iron chelating where the units are mg/ml. Data expressed as mean ± S.D (n = 6). *p < 0.05; **p < 0.01; ***p < 0.001 vs standard.

### Erythrocyte membrane stabilizing activity (EMSA)

**Figure 6:**
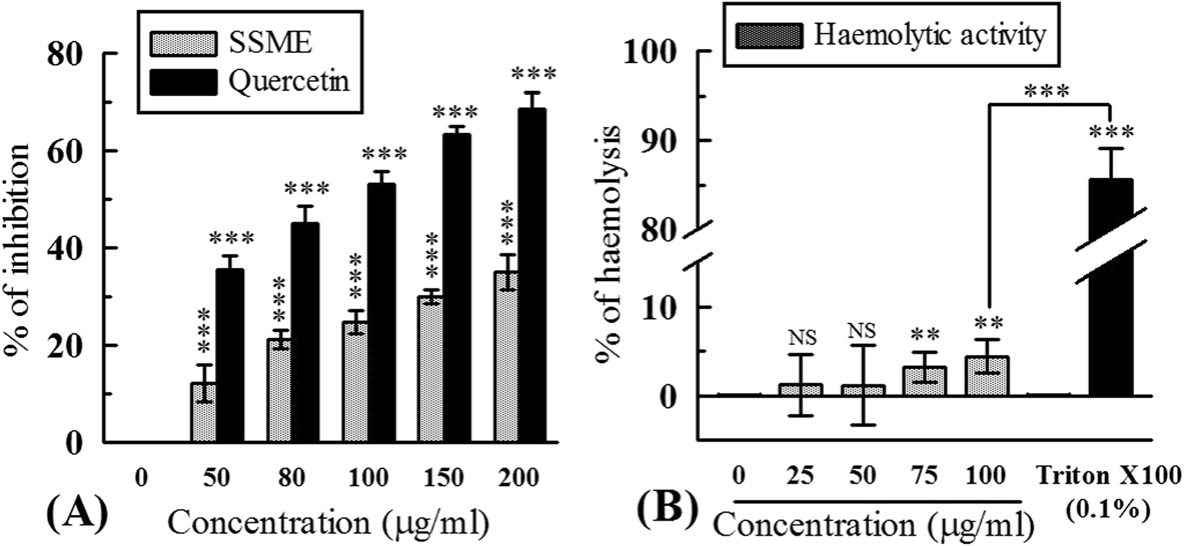
**Bio-activities of SSME.** **(A)** Erythrocyte membrane stabilizing activity; **(B)** Haemolytic activity. ^NS^P > 0.05, **P < 0.01 and ***p < 0.001 vs 0 μg/ml.

Figure 6A displays erythrocyte membrane stabilizing activity of SSME in comparison to standard quercetin. At 200 μg/ml, percentage of protection by SSME was 34.88 ± 3.48% and quercetin was 68.50 ± 3.45%. The half maximal protection efficiency of SSME and quercetin were 341.62 ± 25.73 μg/ml and 91.20 ± 2.16 μg/ml, respectively.

### Haemolytic activity

The haemolytic activity of SSME is displayed in Figure 6B. At 100 μg/ml, the haemolytic activity of SSME was 4.40 ± 1.85% which significantly less than that of triton-X100 (85.63 ± 3.49%). The half maximal haemolytic concentration was calculated to be 2.37 mg/ml.

### Phenolic and flavonoid content

The total phenolic content of SSME was 0.79 ± 0.02 mg/ml GA equivalent/ mg plant extract and flavonoid content was 0.55 ± 0.04 mg/ml QC equivalent per/ mg plant extract.

### HPLC analysis

**Figure 7:**
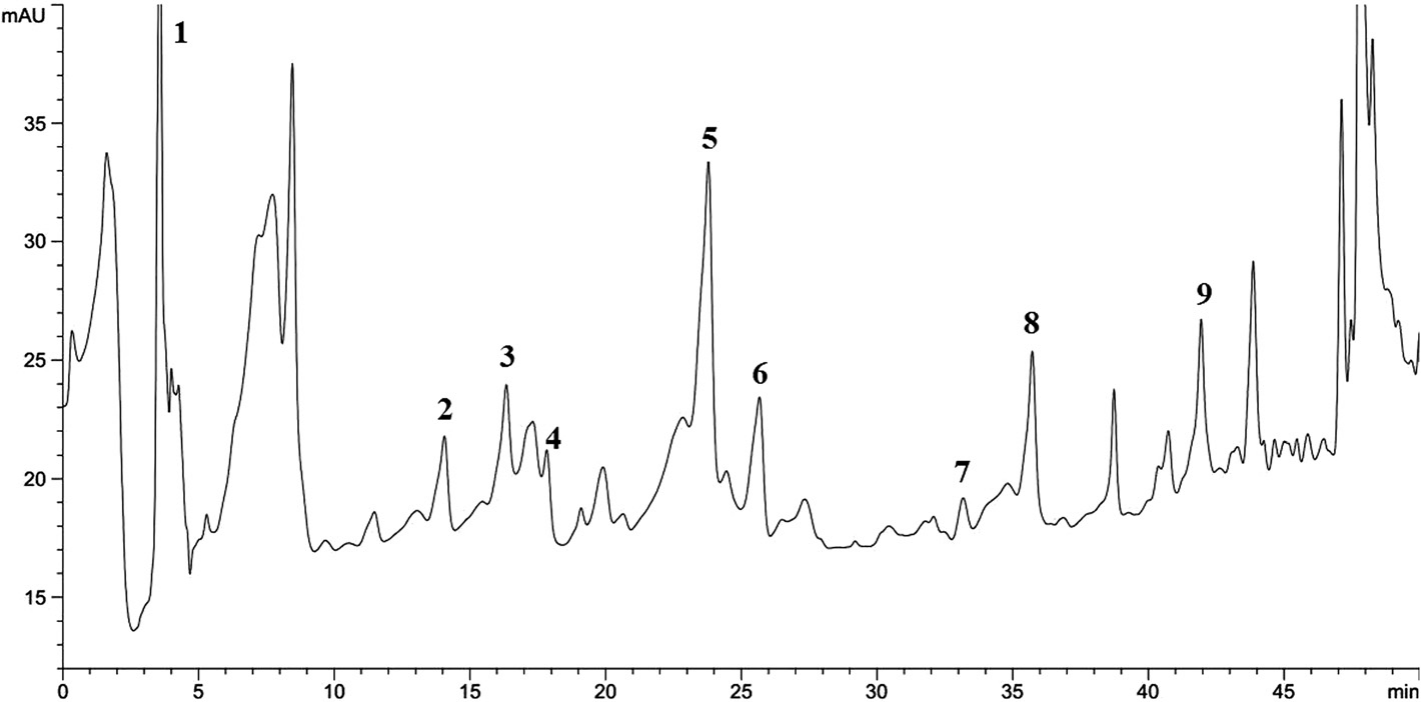
**HPLC analysis of SSME.** Identified compounds at 275 nm were (1) gallic acid, (2) 4-hydroxybenzoic acid, (3) vanillic acid, (4) syringic acid, (5) p-coumaric acid, (6) ferulic acid, (7) p-cresol, (8) myricetin, (9) quercetin.

HPLC analysis of SSME revealed the presence of different bioactive secondary metabolites as represented in Figure 7. Gallic acid (GA; 64 ± 5.10 μg/g dw), 4-hydroxybenzoic acid (4HBA; 3.035 ± 1.09 μg/g dw), vanillic acid (VA; 4.54 ± 1.54 μg/g dw), syringic acid (SA; 16.70 ± 3.15 μg/g dw), p-coumaric acid (PCA; 9.81 ± 3.35 μg/g dw), ferulic acid (FA; 7.98 ± 1.48 μg/g dw), myricetin (MY; 14.14 ± 4.99 μg/g dw) and quercetin (QC; 2.30 ± 0.66 μg/g dw) were the major phenolic acids and p-cresol (PC; 0.57 ± 0.20 μg/g dw) was the soul alcohol identified.

### GC-MS analysis

**Figure 8:**
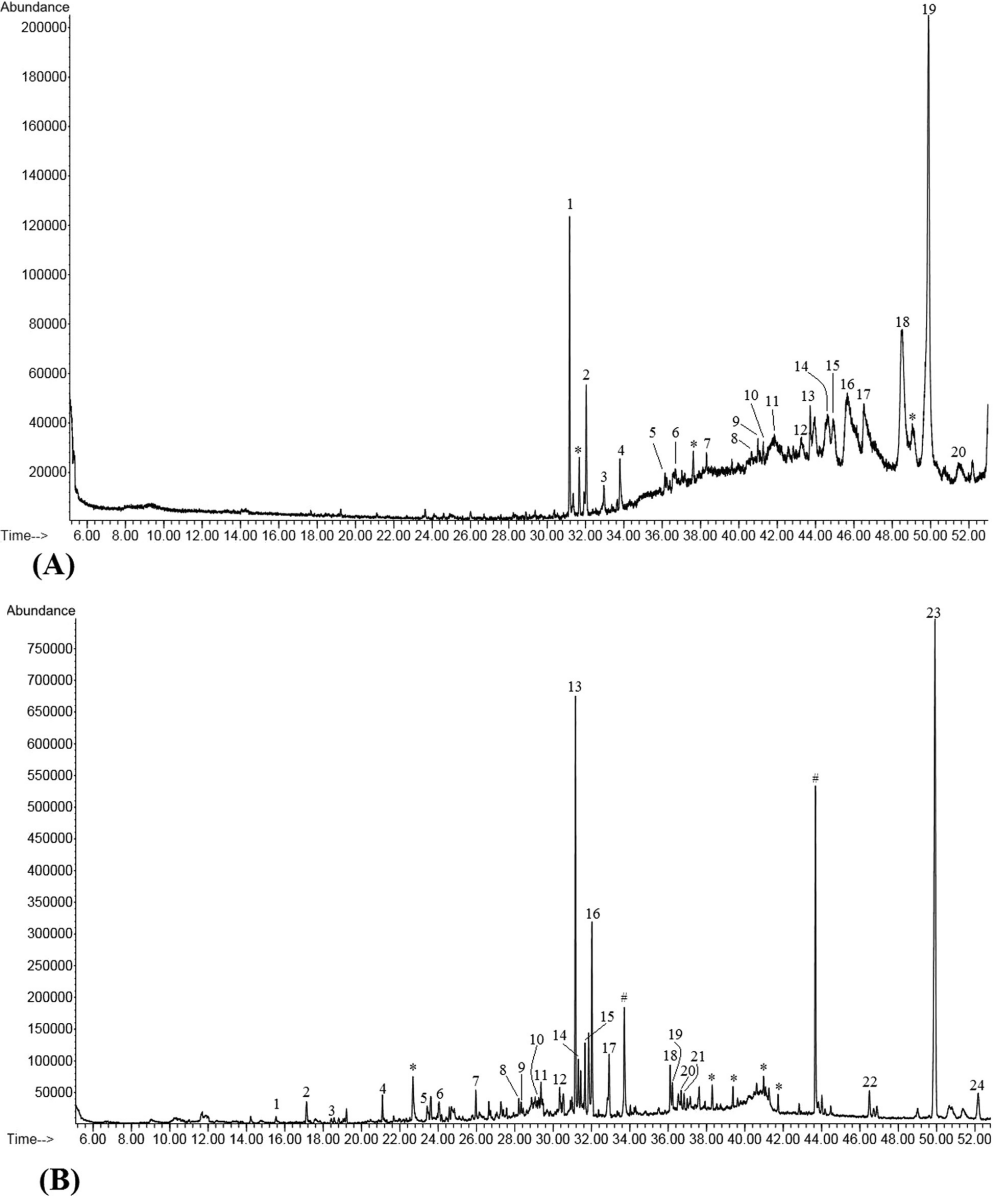
**GC-MS identification of chemical fingerprint of SSME. (A)** SSME dichloromethane fraction. **(B)** SSME n-hexane fraction. * = column component/ repeat compound/ unidentified compound. ^#^ = phthalate compound.

The results of GC-MS analysis is displayed in Figure 8 and Table 2. SSME revealed to contain various bioactive constituents of different chemical nature among which γ-Sitosterol, vitamin E and squalene were predominantly present.

**Table 2:** **List of phytocompounds identified by GCMS analysis which corresponds to Figure** 8

	**SSME dichloromethane fraction (Figure** **8** **A)**		
**SL no.**	**Compound Name**	**Retention time (RT)**	**Relative abundance (%)**
1	Bicyclo [3.1.1] heptane, 2,6,6-trimethyl-, (1α,2β,5α)	31.165	4.17
2	3,7,11,15-Tetramethyl-2-hexadecen-1-ol	32.035	2.13
3	Hexadecanoic acid, methyl ester	32.960	1.01
4	1,2-Benzenedicarboxylic acid, butyl octyl ester	33.799	1.30
5	12-Methyl-E,E-2,13-octadecadien-1-ol	36.144	0.37
6	Methyl trans-9-(2-butylcyclopentyl) nonanoate	36.707	1.23
7	18-Nonadecen-1-ol	38.309	0.53
8	Tetradecanal	40.992	0.54
9	Oxalic acid, cyclobutyl pentadecyl ester	41.268	0.40
10	Hexadecanal	41.568	0.67
11	Octadecenoic acid (Z)-, 2,3-bis (acetyloxy) propyl ester	41.837	2.13
12	Trichloroacetic acid, 3-tetradecyl ester	43.269	1.48
13	Stigmasteryl tosylate	43.957	2.41
14	Stigmastan-6,22-dien, 3,5-dedihydr o-	44.658	3.99
15	Diazoprogesterone	44.914	2.43
16	γ-Sitosterol	45.659	11.95
17	Cholestane, 4,5-epoxy-,(4α, 5α)-	46.522	10.24
18	Vitamin E	48.517	13.74
19	Squalene	49.894	27.34
20	17-Pentatriacontene	51.445	2.03
	**SSME n-hexane fraction (Figure** **8** **B)**		
**SL no.**	**Compound Name**	**Retention time (RT)**	**Relative abundance (%)**
1	Heptane, 2,6-dimethyl-	15.540	0.29
2	Benzene, 1,3-bis(1,1-dimethylethyl)-	17.135	0.60
3	Oxalic acid, isobutyl nonyl ester	18.423	0.15
4	Tetradecane	21.094	0.53
5	Trans-β-Ionone	23.427	0.59
6	Phenol, 2,4-bis(1,1-dimethylethyl)	24.047	0.71
7	Hexadecane	25.967	0.62
8	Heptadecane	28.206	0.48
9	Hexadecane, 2,6,11,15-tetramethyl-	28.332	0.51
10	1-Decanol, 2-hexyl-	29.245	0.63
11	Hentriacontane	29.364	1.42
12	Octadecane	30.340	1.05
13	Bicyclo[3.1.1]heptane, 2,6,6-trimethyl-, (1α,2β,5α)	31.159	6.64
14	2-Pentadecanone, 6,10,14-trimethyl	31.309	1.46
15	1,4-Eicosadiene	31.659	1.94
16	Cyclohexanol, 1-ethynyl-	32.022	3.99
17	Hexadecanoic acid, methyl ester	32.917	1.96
18	9,12-Octadecadienoic acid, methyl ester, (E,E)-	36.107	1.14
19	9-Octadecenoic acid, methyl ester,(E)-	36.213	1.25
20	Phytol	36.526	0.89
21	Heptadecanoic acid, 15-methyl-, methyl ester	36.676	0.71
22	Tritetracontane	46.497	0.75
23	Squalene	49.919	16.10
24	2-methyloctacosane	52.171	1.14

## Discussion

Natural antioxidant and free radical scavengers are the current focus in nutrition and health supplements besides most plants and polyherbal formulations of the traditional medicinal system are found to possess potent antioxidant capacities [32]. In recent years, the phrase ‘oxidative stress’ has become a potential concern in medicine which is reflected by the fact that the antioxidant related publications has nearly quadrupled in the past decade [33]. Oxidative stress, which is primarily generated due to the imbalance between reactive oxygen species (ROS) and antioxidative protection, going in favor of the former, is responsible for most of the major diseases. In addition to oxidation of polyenoic fatty acid rich foods, adverse environmental conditions such as pollution and UV radiation generates a plethora of free radicals which eventually mediates the degradation of cellular biomolecules.

Reducing power can be attributed to the anti-ROS activity as oxidation is the predominant mechanism behind free radical medicated damage even though a potent antioxidant should possess individual free radical scavenging properties besides potentials to chelate metals and inhibit lipid peroxidation [34]. TEAC and DPPH assay are the two most commonly used electron transfer methods for the assessment of antioxidant capacities of natural products. The concentration of test material giving same percentage of inhibition of ABTS^•-^ compared to the same of trolox is regarded as TEAC. The ABTS^•-^ scavenging capacity of SSME was compared to that of trolox which demonstrated convincing TEAC value (0.81 ± 0.006). Similarly, DPPH which is a stable organic nitrogenous radical, [33] was scavenged by SSME at very low concentration. The DPPH scavenging activity of SSME was in fact higher than that of ascorbic acid.

Though short lived and low in concentration, hydroxyl radical (OH^•^) is an extremely harmful ROS with tremendous potentiality to damage biomolecules [33]. OH^•^ is commonly generated through the Fenton reaction between Fe^2+^ and H_2_O_2_ [35]. On the contrary, metal chelators may also contribute to the reduction of OH^•^ by means of conversion of Fe^2+^ to Fe^3+^ and thus, hindering the Fenton reaction. Besides, breakdown of H_2_O_2_ into O_2_ and H_2_O may also prevent OH^•^ formation [33]. H_2_O_2_ being a potent oxidizing agent by itself can affect cellular enzymes by oxidation of essential thiol (−SH) groups [19]. Excess iron (Fe^2+^) may further catalyse superoxide anion (O^•2−^) mediated Haber-Weiss reaction to generate OH^•^ which ultimately results in lipid peroxidation. Highly toxic superoxide anion (O^•2−^) is primarily generated from the mitochondrial respiration and its spontaneous dismutation may initiates lipid peroxidation by generation of singlet oxygen (^1^O_2_) [36]. ^1^O_2_ on the other hand, is responsible for UV dependent skin damage and it initiates cardiovascular disorders by oxidation of LDL cholesterol. Various inflammatory conditions and carcinomas are associated with the binary molecule NO which itself couples with O^•2−^ to generate peroxynitrite (ONOO^−^) [33]. ONOO^−^ and its protonated more reactive form ONOOH often results in nitration or hydroxylation of aromatic compounds and forms abduct with CO_2_ which in turn responsible for oxidative damage of proteins [36,37]. Myeloperoxidase-mediated peroxidation of chloride in neutrophils gives rise to hypochlorous acid which mediates irreversible oxidation to cellular components [38]. Furthermore, various ROS such as OH^•^ and H_2_O_2_ initiates a self-propagating chain reaction by attacking PUFA and thus, cause lipid peroxidation, responsible for pathogenesis of various diseases [39].

Previously Nguyen and Eun [5] studied the antioxidant capacity of different solvent extracts of *Streptocaulon juventas*, among which the aqueous extract demonstrated DPPH radical scavenging activity of 44.07 ± 0.07% (DPPH:extract = 1:3, v/v) besides potent reducing power (IC_50_ 0.85 ± 0.02 mg/ml). The iron chelation capacity was higher in methanolic extract with an IC_50_ value of 1.67 ± 0.04 mg/ml. In addition, the acetone and aqueous extracts respectively contained highest phenolic (93.22 ± 0.10 mg GA equivalent/g extract) and flavonoid (58.09 ± 0.14 mg QC equivalent per g extract). On the other hand, antioxidant property of *Streptocaulon boumi* was evaluated using a modified DPPH assay which demonstrated −4.3% DPPH scavenging activity compared to GA [6]. In the present study SSME was evaluated for its antioxidant and free radical scavenging potentials through different antioxidant assays. SSME has demonstrated excellent OH^•^, O_2_^•-^ and HOCl scavenging activity, better than the respective standards. Besides, though SSME failed to display a convincing H_2_O_2_ activity, its iron chelation capacity was potent enough which may inhibit OH^•^ generation by inhibiting catalysis of Fenton reaction.

The erythrocyte ghost is extremely susceptible to oxidative damage affecting the deformability, aggregability of the cells. External superoxide radical (O_2_^•−^) mediated attack gives rise to change in the dynamics of blood flow [40]. Erythrocyte membrane degradation in sickle condition is also governed by ROS mediated attack [41]. In such conditions the production of O_2_^•−^, OH^•^ and peroxide are elevated by two fold, thus requiring protection of the membrane from free radicals. In EMSA, haemolysis of erythrocytes was induced by generation of O_2_^•−^ through autoxidation of riboflavin in presence of light. SSME displayed excellent membrane stabilizing capacity by inhibition of superoxide radical. Besides it has also displayed *in vitro* O_2_^•−^ scavenging activity which may further be an aid in erythrocyte membrane protection.

Haemolytic activity is one of the parameters of cytotoxicity by natural compounds. Certain ingredients in dietary supplements often contain varying amount of haemolytic activity such as saponins from alfalfa [42] results in release of haemoglobin due to irreversible damage of erythrocyte membrane. Winter (1994) described the process of saponin induced haemolysis mediated by increase of water carriage by aquaporins [43]. At the highest dose even though compared to control SSME displayed statistically significant (P < 0.01) Haemolytic activity, but considering such high HC_50_ value the activity may be considered negligible. Besides, the haemolytic activity of SSME was significantly (P < 0.001) less than that of the standard triton-X100.

Today, the inventory of antioxidative phytochemicals chiefly consist of phenolic and flavonoid compounds which have also demonstrated a vast range of bioactivities. Antioxidant properties of phenolics are governed by their ability to be oxidized apart from their capacity to turn hydroxyl radicals into harmless OH^•^ derivative through incorporation into double bonds. The HPLC analysis revealed different bioactive secondary metabolites which have already been recognized for their pharmacological properties. GA [44], VA [45], FA [46], PCA [47], MY and QC [48] possess potent antioxidant capacities. Liu *et al*. [49] previously isolated FA, SA and β-sitosterol from *S. juventas*. In addition, GC-MS analysis revealed the presence of very high quantity of γ-sitosterol, vitamin E and squalene. Sitosterol possess anti-diabetic and free radical scavenging capacity [50]. Squalene and vitamin E are also predominant among the phytoconstituents of some other plants of the same family (Apocynaceae) [51]. Apart from its active role in nutrition, vitamin E is a natural antioxidant. Further, squalene is also a natural antioxidant found in majority of plants [52]. Therefore, the presence of these bioactive phytochemicals in *S. sylvestre* is the responsible factors behind the potent antioxidant activity of SSME.

## Conclusion

The current study reports the first ever pharmacological evaluation of a rare plant *S. sylvestre* in addition to elucidation of its chemical fingerprints. In recent years, free radical mediated oxidative stress has emerged as the corner-stone of the pathogenesis of several harmful diseases [18]. NO, ONOO^−^, H_2_O_2_, O_2_^•−^ are OH^•^ are essential parts of immune reactions in phagocytes during chronic pro-inflammatory response, causing tissue damage. Moreover, progression of diabetic complications [53] and hepatotoxicity due to idiosyncratic drug reaction [54] are primarily governed by free radicals, reactive metabolic intermediates and subsequent depletion in antioxidative protection. Therefore, the antioxidant capacity of *S. sylvestre* may prove beneficial in amelioration of such conditions. *S. sylvestre* also holds tremendous potential for its therapeutic efficiency in future due to presence of several bioactive constituents. However, at present awareness of the existence of the plant and plant tissue culture based methods are required for further propagation of this rare plant in different parts of the world.

## Additional file

Additional file 1:
**Supplementary data.**
