# Supplementary figures and images for: Chemical characterization and assessment of antioxidant potentiality of Streptocaulon sylvestre Wight, an endangered plant of sub-Himalayan plains of West Bengal and Sikkim
Source: BMC Complement Altern Med. 2015 Apr 8;15:107. doi: 10.1186/s12906-015-0629-0 (PMC4396540; doi:10.1186/s12906-015-0629-0)

**Supplementary data 3**


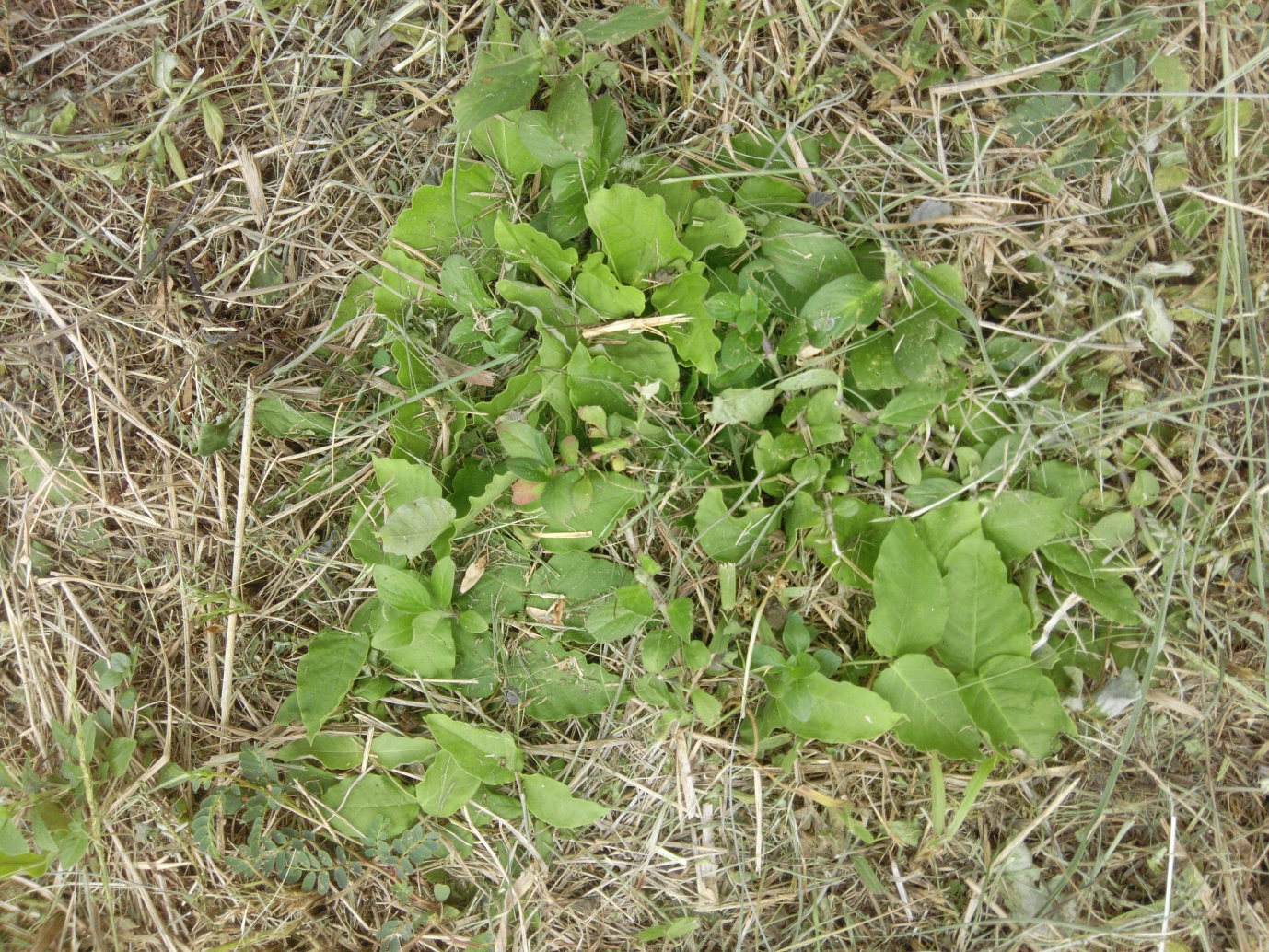


One of the patch of *S. sylvestre.*


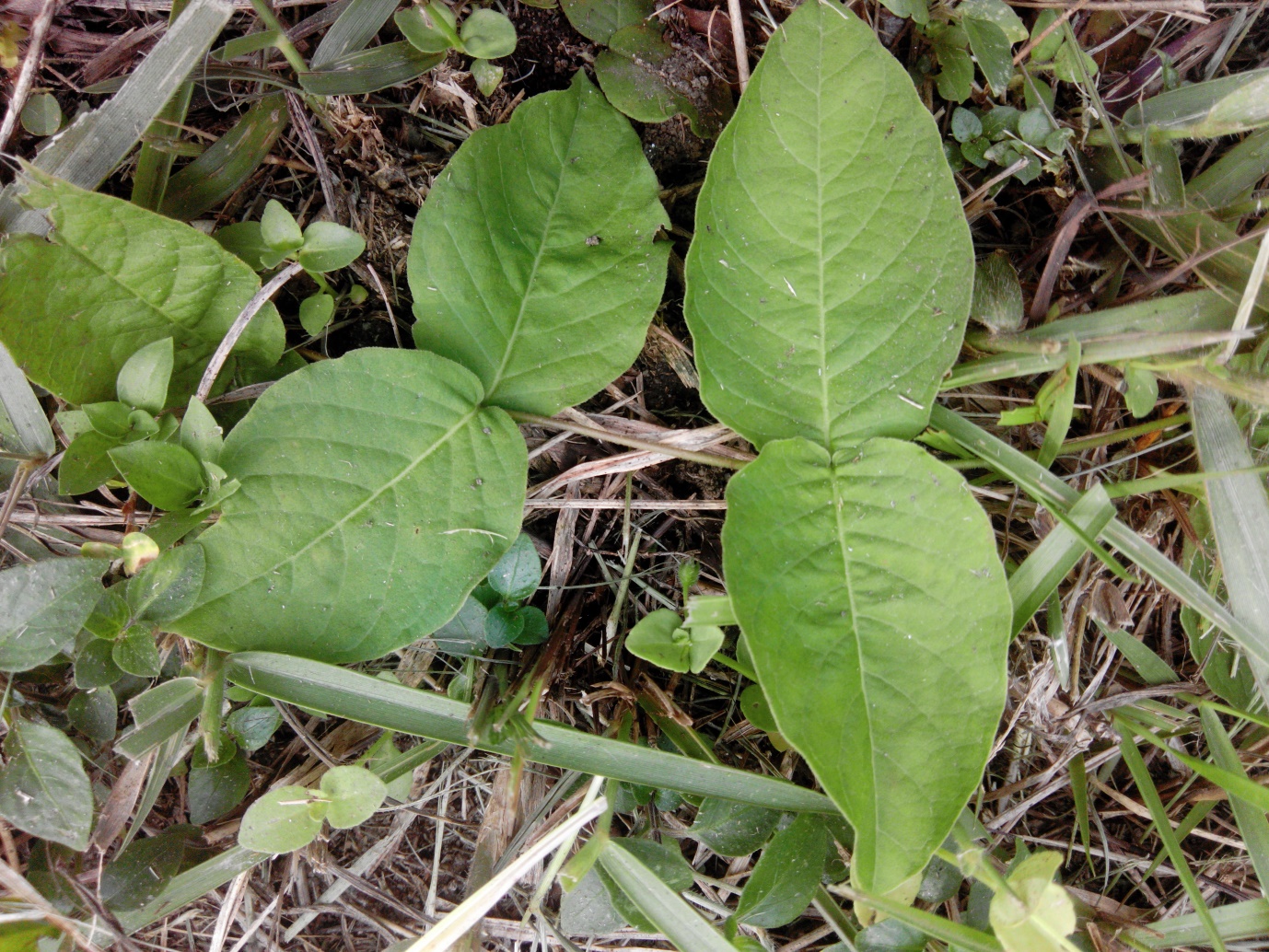


Closer view of the leaves of *S. sylvestre.*

Supplement: Additional file 1: — Supplementary data. [file 12906_2015_629_MOESM1_ESM.docx]
